# Individually Specific Call Feature Is Not Used to Neighbour-Stranger Discrimination: The Corncrake Case

**DOI:** 10.1371/journal.pone.0104031

**Published:** 2014-08-04

**Authors:** Michał Budka, Tomasz S. Osiejuk

**Affiliations:** Department of Behavioural Ecology, Institute of Environmental Biology, Faculty of Biology, Adam Mickiewicz University, Poznań, Poland; University of London, United Kingdom

## Abstract

In various contexts, animals rely on acoustic signals to differentiate between conspecifics. Currently, studies examining vocal signatures use two main approaches. In the first approach, researchers search for acoustic characteristics that have the potential to be individual specific. This approach yields information on variation in signal parameters both within and between individuals and generates practical tools that can be used in population monitoring. In the second approach, playback experiments with natural calls are conducted to discern whether animals are capable of discriminating among the vocal signatures of different individuals. However, both approaches do not reveal the exact signal characteristics that are being used in the discrimination process. In this study, we tested whether an individual-specific call characteristic – namely the length of the intervals between successive maximal amplitude peaks within syllables (PPD) – is crucial in neighbour-stranger discrimination by males of the nocturnal and highly secretive bird species, the corncrake (*Crex crex*). We conducted paired playback experiments in which corncrakes (n = 47) were exposed to artificial calls with PPD characteristics of neighbour and stranger birds. These artificial calls differed only in PPD structure. The calls were broadcast from a speaker, and we recorded the birds' behavioural responses. Although corncrakes have previously been experimentally shown to discriminate between neighbours and strangers, we found no difference in the responses to the artificial calls representing neighbours versus strangers. This finding demonstrates that even if vocal signatures are individual specific within a species, it does not automatically mean that said signatures are being crucial in discrimination among individuals. At the same time, the birds' aggressive responses to the artificial calls indicated that the information transmitted by PPDs is important in species-specific call recognition and may be used by males and/or females to evaluate sender quality, similarly like sound frequency in some insect species.

## Introduction

Animals commonly use acoustic signals to discriminate between different classes of individuals or to accurately identify individuals. In various vertebrate species, such as amphibians, reptiles, birds and mammals, songs or calls can be used to identify parents [1], [2], mates [3]–[5], group members [6], [7], kin [8] and territorial neighbours and strangers [9]–[11]. Irrespective of the taxon being considered or the context in which the vocalisation is being used, two main assumptions must be met for discrimination to be possible: (1) acoustic signals must have at least one characteristic that is specific to individuals, meaning that it demonstrates far less within- than between-individual variation [12], [13] and (2) individuals should be able to perceive and remember how individuals differ with regards to this signal characteristic [14], [15].

Currently, studies of vocal signatures employ two main approaches. In the first, the potential of different signal characteristics to play a role in individual vocal identity is considered from a sender perspective. Researchers simply explore within- and between-individual variation in many characteristics of a species' call or song and suggest that those characteristics with low within- and high between-individual variation could be used to differentiate among individuals [16]–[19]. This approach may yield tools that are helpful to species conservation and monitoring efforts, especially when dealing with species that are difficult to observe visually because they live in dense habitats or/and are active at night [20], [21]. Furthermore, individual-specific vocalisations may be used to track individuals, offering a non-invasive alternative to more intrusive approaches such as mark-recapture techniques or tagging [22]. However, although individuals may demonstrate unique vocal characteristics, this does not mean that such characteristics are used by birds to identify different individuals. In the second approach vocal individuality is considered from the receiver's perspective. Then researchers commonly apply playback experiments, in which the natural calls or songs of known and unknown individuals are broadcast from a speaker [9], [10], [23], [24]. This approach enables researchers to definitively confirm that acoustic discrimination is taking place in a particular species. However, the signal characteristics being used in this discrimination process are not clarified by the experiments.

When a species' ability to acoustically discriminate among conspecifics has been experimentally confirmed and the call characteristics that have the potential to be individual specific have been identified, playback experiments employing manipulated call features may reveal which signal component is used in the acoustic discrimination process [25], [26]. In particular, it is the differences in the subjects' responses to the modified signal characteristics that suggest that specific call characteristics are involved [25], [27].

**Figure 1:**
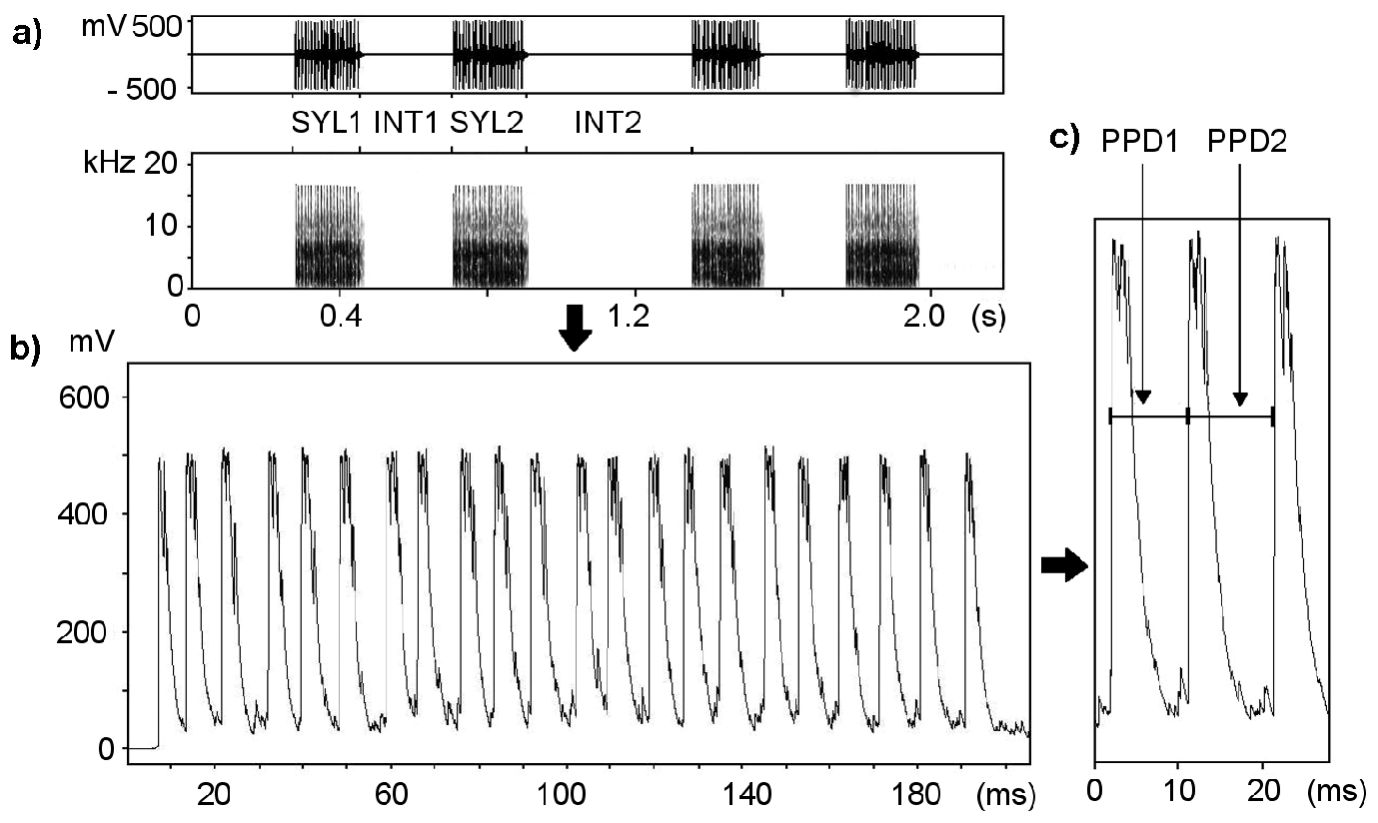
Typical corncrake call. Each call consists of first (SYL1) and second syllable (SYL2), within- (INT1) and between-call interval (INT2) (a). Each syllable usually consists of 14–22 repeated maximal amplitude peaks (b). The duration of the intervals between the successive maximal amplitude peaks within the syllables (PPD 1, PPD 2, etc.) are individual specific and constant throughout a bird's life (c).

**Figure 2:**
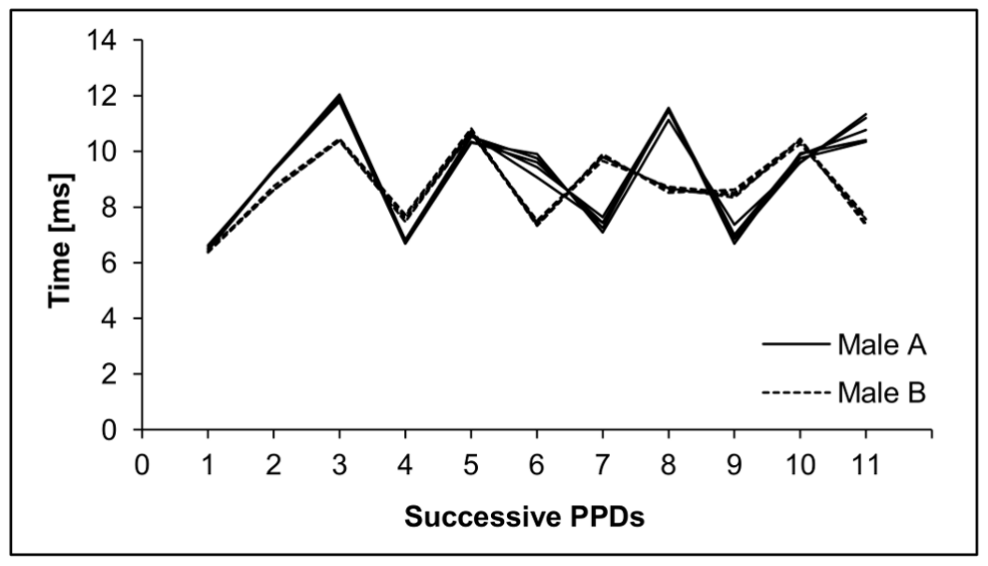
Differences between the first eleven PPDs of two randomly selected males from Kampinoski National Park. Five calls from Male A and five calls from Male B are shown. The graph shows small within- and large between-individuals variation in PPD.

In this study, we focused on acoustic discrimination between neighbours and strangers in a model bird species - the corncrake, *Crex crex*. Corncrakes inhabit wet meadows that are characterised by dense vegetation, and thus visual contact among individuals is very limited [28], [29]. Males are vocally active at night: they make a characteristic monotonous and loud cracking call [30]. The call has a simple structure; it consists of two syllables (SYL1 and SYL2) that are separated by an intervening interval (INT1) (Fig. 1). The syllables are structurally toneless and usually contain 14 to 22 repeated amplitude peaks. The duration of the intervals between successive maximal amplitude peaks within a syllable, which is called the pulse-to-pulse duration (PPD), remains constant throughout a bird's life. As a result, males can be distinguished based on this call characteristic (Fig. 2) [17], and PPD has previously been used to identify males [21], [31]. Moreover, it has been experimentally shown that corncrake males can discriminate between neighbours and strangers based on their calls. When a stranger's call is played back, males approach the speaker more quickly, spend more time in the vicinity of the speaker, and physically attack the speaker more often than if a neighbour's call is played [9]. This behaviour is in accordance with Fisher's dear-enemy rule [32]. The corncrake's aforementioned ecology and communication system thus make it an ideal model with which to study the mechanisms of acoustic discrimination.

More specifically, we experimentally examined if corncrake males use an individual-specific call characteristic, PPD, to discriminate between neighbours and strangers. We conducted a playback experiment in which we broadcast calls from a speaker (artificial calls with PPD characteristics of neighbour and stranger birds) and then quantified the birds' responses to the calls. We predicted that if PPD is crucial to discriminate between neighbours and strangers in corncrake, then they would react differently towards the calls of neighbours versus those of strangers, as has been previously observed in a similar study [9].

## Methods

### Study area and call recording

The study was conducted in Kampinoski National Park (N 52.325° E 20.510°; central Poland) from May 23 to June 7, 2011. The study area is an open complex of swamps, abandoned and extensively mowed meadows. Corncrake males are irregularly distributed within the study area, and it is estimated that the population contains between 110 and 140 calling males [33]. Our study birds were not marked but rather were identified based on their PPDs [17]. The calls of neighbouring birds were recorded one to three days before each playback session. The strangers' calls were recordings we had made in Kampinoski National Park in 2010. All recordings were collected at night (from 2200 to 0400 hours, local time) and at a distance of 5–15 m from the calling bird. We used Edirol R-09 or Marantz PMD 620 recorders and a Sennheiser ME-67 directional microphone with a K6 power module. The position of the calling male was always quantified using GPS. All recordings were of the same digital quality (44.1 kHz/16 bit).

### Preparation of artificial call stimuli

In our playback sessions, we used artificial calls that differed only in PPD. We developed a unique set of artificial calls (one from a neighbour and one from a stranger) for each study bird. To do so, we first measured PPD and pulse duration in two randomly selected calls (CALL = SYL1 + INT1 + SYL2) (Fig. 1) made by a neighbour and a stranger using Avisoft SASLab Pro version 5.0.16 [34]. The general settings were as follows: FFT = 1024, frame = 25%, window = hamming, and temporal overlap = 98.43%. PPD structure was analysed using the “pulse train analysis” function. Before measuring PPD, we used the FIR time-domain filter (500 Hz; high pass setting) to remove low-frequency noises from all of the sound files. In the pulse train analysis, we used the “rectification + exponential decay” method to measure PPD and employed the following settings: time constant = 1 ms, threshold = 0.10 V, hysteresis = 10 dB, and start-end threshold = −8 dB. All pulse distribution measurements were visually checked to confirm that all of the pulses have been detected.

We then constructed the artificial calls in Adobe Audition 1.0 software. First, we started by adding a few seconds of silence. Then we generated white noises of a given duration and interval between them, which were characteristic for a mimicked individual. White noise is a signal that contains the same amount of energy on every frequency of the spectrum. The duration of a first white noise was the same as the duration of the first pulse within the mimicked syllable. A second white noise was then added; its distance from the first white noise was the same as the distance between the syllable's first and second pulse. The duration of a second white noise was the same as the duration of the second pulse within mimicked syllable. In this way we added successive white noises, and we ultimately ended up with an entire artificial syllable that had the same white noises distribution as PPD distribution within the original syllable. Finally we got artificial calls (comprising the two first and second syllables of a neighbour and a stranger call) with the same acoustic energy in each frequency range. In the corncrake, the first 10–14 pulses within each syllable remain constant throughout the male's life [17]. In our study population, the minimum number of pulses within each syllable was 12. To compare the artificial and original call structures, we calculated the Pearson correlation coefficients between the first 11 PPDs of the original and artificial syllables. In all cases, they were highly correlated (r>0.90). Such within-individual variation is observed within corncrake males' vocalization [17].

In the corncrake, the distribution of acoustic energy across the call frequency range is varied [35]. We therefore filtered our artificial calls using the graphic equalizer filter (30 bands, 1/3 octave) in Adobe Audition. To determine the appropriate filter parameters, we measured the distribution of energy across the frequency range of calls obtained from 55 corncrake males recorded in 2010 in Kampinoski National Park (20 calls from each male). We measured minimal (MINF) and maximal (MAXF) frequency as well as the frequencies below which 25% (L25), 50% (M50) and 75% (U75) of the total energy of the acoustic signal was found. The means and standard deviations (kHz) of these variables were as follows: MINF = 1.2±0.31; MAXF = 8.1±0.83; L25 = 3.4±0.46; M50 = 5.0±0.44; and U75 = 6.1±0.35. We used these values to set filter parameters so that the energy distribution (mean ± SD) of the artificial calls resembled that of the population. The same filter settings were applied to each artificial call. The syllable and interval durations were exactly the same in the artificial calls representing neighbours versus strangers. The amplitude of each broadcast call was set to have a signal pressure level of 95±5 dB (at 1 m), which is the average amplitude for the natural calls of corncrake males [29]. Examples of natural call (Call S1), artificial call with the same acoustic energy in each frequency range (Call S2) and artificial call filtered by graphic equalizer filter (Call S3) are included as supporting information.

### Experimental procedure

In our playback experiment we used 47 corncrake males. The playback sessions were conducted at night (from 2230 to 0330) and during good weather conditions (without rain or strong wind). We broadcast calls from a speaker (SEKAKU WA-320, Taichung, ROC Taiwan; 20 W amplifier and a frequency range of 50–15,000 Hz) connected to a Creative ZEN player. Birds were considered to be neighbours if they had been recorded calling the same year and at a distance of less than 300 m from each other. Birds were considered strangers if they had been recorded calling in different years and more than 5 km apart. In this way we reduced the probability virtually to zero that a tested and a stranger male were in the contact in the past.

Each bird experienced two playback trials, one involving a neighbour-like call and one involving a stranger-like call. The order in which the call types (neighbour vs. stranger) were played was randomly determined for each bird. In the first trial, the speaker was placed on top of a plastic box (0.5 m above ground level) and was situated between the study bird and the neighbour whose call had been used; the speaker was approximately 20 m from the study bird. In the second trial, the speaker was once again placed between the study bird and his neighbour, approximately 20 m away, but in a slightly different position (usually at a distance of 20–30 m from the previous location). The time between trials ranged from 1 to 4 hours and was long enough to allow the birds to return to their pre-stimulus levels of behaviour. The male neighbour whose call had been used was kept quiet during both trials. Field assistants approached him and made noise, which caused him to go silent. We used calls from 47 different neighbours and 47 different strangers to avoid pseudoreplication. The person observing the study bird's behaviour did not know which type of call (neighbour vs. stranger) was being broadcast during the trial.

We used the same playback procedure as in our study examining how corncrakes discriminate between neighbours and strangers [9]. Each trial consisted of two phases. First, the male's behaviour was recorded for one minute before playback occurred. Second, the male's behaviour was recorded for five minutes as playback occurred. The call was broadcast as soon as the study bird became vocally active, and the broadcast ended 10 seconds after the bird stopped calling. Playback was reinitiated only if the bird started calling again. As a result, call playback duration ranged from 10 seconds to 5 minutes. This type of experimental approach, in which an individual's behaviour determines playback length, is representative of natural interactions between real rivals and has previously been successfully used in playback experiments involving corncrakes [9], [36].

### Response measures and statistical analyses

During the experiment, we noted the following responses: (1) the time it took the bird to first approach the speaker (5-m radius), (2) the total amount of time spent within 5 m of the speaker, and (3) the number of times the bird attacked the speaker. These same behavioural responses were quantified in an earlier study and found to unambiguously reflect differences in how birds respond to neighbours versus strangers. Furthermore, these responses are easy to measure even at night. Before the experiment started, a 5-m radius was traced around the speaker, and the observer could focus on the bird's movements towards and within this space. It was also easy to hear the sound of the male attacking the speaker's plastic pedestal.

The birds' responses to the different call types were analysed using generalized estimating equations (GEE). Our dependent variables (time to first approach, time spent within 5-m of the speaker and frequency of attacks directed at the speaker) were binomial (with values of 0 or 1). If the variable had a value of 1, it indicated that the bird got within 5 m of the speaker more quickly, spent more time within 5 m of the speaker, and attacked the speaker more frequently, respectively. We therefore fit the data to the models using a binomial distribution (logit link function). Our categorical predictor variables were call type (neighbour or stranger) and playback order (neighbour first or stranger first). All of the statistical analyses were run in IBM SPSS Statistics 21. All the p-values were two tailed.

### Ethics

All experimental procedures were conducted in accordance with the ARRIVE guidelines for animal research [37]. This study fully complied with the current laws of Poland. Our research was approved by the Poznań Local Ethics Committee for Animal Experimentation (decision no. 31/2011). This project was also approved by the General Directorate for Environmental Protection in Poland (permit no. DOP-OZGIZ.6401.03.190.2011.dl). We had a permit from Director of Kampinoski National Park to conduct the study in Kampinoski National Park.

## Results

**Figure 3:**
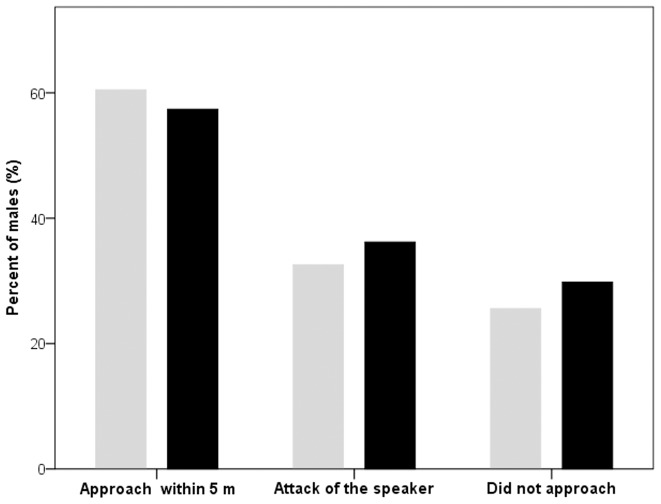
Intensity of response to playback with natural calls (grey bars; n = 43) and with artificial calls (black bars; n = 47). Percent of males which approached within 5(artificial calls) and our previous study of corncrake neighbour-stranger discrimination (natural calls) [9]. Treatments of neighbours and strangers were considered together. Response was counted when male reacted at least in one of the treatments.

We examined 47 territorial males. Overall, 27 of the 47 males we studied got within 5 m of the speaker and 17 males attacked the speaker at least once. When a neighbour-like call was being broadcast, 29 males approached the speaker, 22 of them got within 5 m of it, and 9 attacked it. When a stranger-like call was being broadcast, 27 males approached the speaker, 22 of them got within 5 m of it, and 10 attacked it. For more details see table 1 and figure 3.

**Table 1:** Measures of responses to playback with the artificial calls of a neighbour and a stranger by Corncrakes' males.

Respond measures	N	Neighbour-like stimulus	Stranger-like stimulus
Latency to first approach within 5 m (s)	22	146±81.2 (59±78.4)	111±75.2 (90±95.0)
Total time within 5 m of speaker (s)	47	72±93.7 (50±69.0)	75±95.7 (85±92.7)
Number of attacks of the speaker	47	0.83±2.59 (0.16±0.57)	1.47±3.85 (0.51±0.91)

Values are mean ± standard deviation. The latency to approach to a distance less than 5 m from the speaker was transformed by substracting the original values from the maximum possible value (300 s). Thus higher values indicate a more rapid approach. In brackets responses to natural calls from a previous study of corncrake neighbour-stranger discrimination are given [9].

We found no significant differences in how corncrakes responded to artificial calls with PPD characteristics of neighbours-like versus strangers-like birds (Table 2). The males we studied took the same amount of time to approach the speaker, spent the same amount of time around the speaker, and attacked the speaker with the same frequency, regardless of call type. Playback order (neighbour first vs. stranger first) had no significant impact on the response behaviour.

**Table 2:** Results of the generalised estimating equations showing differences in responses towards artificial calls with PPD characteristics of neighbour and stranger males.

Dependent variable	Frequency of attacks directed at the speaker		Time taken to approach the speaker (5-m radius)		Time spent within 5 m of the speaker	
	Wald *χ^2^*	*P (df = 1)*	Wald *χ^2^*	*P (df = 1)*	Wald *χ^2^*	*P (df = 1)*
Intercept	39.05	0.001	26.52	0.001	24.88	0.001
Treatment	2.24	0.134	0.34	0.561	0.58	0.446
Sequence	0.30	0.586	0.04	0.837	0.16	0.693
QIC/QICC	89.0/88.8		119.0/118.2		120.3/119.5	

The models included treatment (neighbour vs. stranger) and playback order (neighbour first vs. stranger first) as independent variables. Dependent variables were expressed on a binomial scale (values of 0 or 1): a value of 1 indicated that the bird attacked the speaker more frequently, got within 5 m of the speaker more quickly, or spent more time within 5 m of the speaker. The Wald statistics and P-values are given. OIC – the quasi-likelihood under independence model criterion; QICC – the corrected quasi-likelihood under independence model criterion.

## Discussion

Playback experiments utilising completely artificial calls are only rarely used in studies of animal acoustics [38], [39]. However, it is one of the most effective and reliable approaches that allow researchers to control all aspects of the acoustic signal; they manipulate only the feature that is of interest. The relatively simple structure of corncrake call enabled preparation completely artificial stimulus. We assumed that a corncrake's identity is revealed acoustically by its PPD, since this trait is constant throughout a bird's life and demonstrates markedly less variation within individuals than between individuals [17]; furthermore, PPDs, and thus the information they encode, are readily transmitted in the natural environment [40]. Therefore we generated artificial calls that had PPD similar like in natural call of neighbour and stranger. The energy distribution across frequency range in our stimulus was average for studied population and constant for neighbour-like and stranger-like call samples. Tested birds were familiar with PPD structure of their neighbours, since they heard neighbours' call across the breeding season. Simultaneously they were unfamiliar with PPD structure of strangers, since the probability that tested bird and stranger bird have met in the past was marginal. Thus in our experiment we were able to examine whether PPD structure is crucial in NSD.

When artificial calls were broadcast from a speaker, males were able to recognise them as coming from members of their own species. Similar proportions of males approached or attacked the speaker in this study and in our previous study of corncrake neighbour-stranger discrimination, a study in which we used natural calls produced by neighbours and strangers (Fig. 3) [9]. In the present study, however, male corncrakes did not appear to respond differently to artificial calls containing the PPDs of neighbours versus strangers: they were equally aggressive in both cases. Lack of differences in response is rather unlikely to be an effect of the speaker placement [41]. In our previous study [9] natural calls were broadcasted from the speaker which was also placed approximately 20 m from the subject and we found significant differences in response to intrusions of neighbours and strangers. The present finding, which is based on a large sample size (n = 47 males), demonstrates that an acoustic feature which expresses individual identity (in this case PPD) [17] is not sufficient when it comes to discriminating among conspecifics. At the same time, the aggressive responses prompted by the artificial calls show that they are good imitations of natural calls and that males recognized them as coming from conspecifics. As a result, the information conveyed by PPDs is likely nonetheless important in species recognition processes, and probably PPD in combination with other call parameters, like call spectral characteristics, can be important in individual identification. Nevertheless, PPD is not crucial in individual identification.

Animals produce sounds within a species-specific range and transmit acoustic information in various ways, including via sound frequency, amplitude, and duration. Particular species are good receivers only in those characteristics of sound which are used to effective communication [42]. Therefore, some species can detect even small dissimilarities in frequency, amplitude, or temporal resolution, while others can only pick up on larger differences [15]. The ability of PPDs to reveal corncrake identity is based on the fact that a specific combination of shorter and longer PPDs is found within each syllable [17]. The intervals between successive pulses (PPD) are really short (on average 7–13 ms; see Fig. 2). Pulses last approximately 3–5 ms. As a result, the duration of the interval between the end of one pulse and the beginning of the next is about 4–8 ms [17]. Birds are able to distinguish between two sounds when the interval between them is at least 1–4 ms [43]. Therefore, males should theoretically experience each pulse as a separate sound. However, to distinguish between conspecifics using PPDs, corncrakes must be capable of perceiving small differences in pulse duration (0–2 ms) and in the intervals that separate them (0–4 ms). It may be difficult to detect and process such minute differences if organisms lack supplementary neuroanatomical adaptations, such as those seen in cetaceans [44] or swiftlets [45], both of which use acoustic communication and echolocation. It thus appears that acoustic recognition by corncrake males does not simply rely on temporally coded information, such as the information expressed by the distribution of shorter and longer pulses and their intervening intervals (which resembles Morse code). In contrast, males may employ spectral characteristics to identify conspecifics. It has already been shown that spectral characteristics, otherwise known as formant frequencies, may be highly individual specific in corncrakes [16]. As a consequence, corncrakes may use formant frequencies to discriminate among conspecifics, similarly as occurs in mammals [46].

The results of this study allow us to better understand the multidimensional acoustic communication in corncrakes, where individuals transmit various types of information to different kinds of receivers at the same time. The corncrake is a species in which calls are generally not learned but rather inherited [47]. However, males are able to change between-call intervals and calling rhythm over a very short period of time [9], [48]. Moreover, calling rhythm is a type of conventional signal that is used by males to signal aggressive motivation [48], [49] and the meaning of this signal is learned during social interactions [50]. In short-distance communication, senders signal their aggressiveness by uttering soft calls [36]. The body size of the sender may be communicated by formant frequencies, because formant frequencies are weakly but significantly correlated with body size in the corncrake [16]. Unfortunately, we do not yet know which call characteristics are used by females to evaluate male quality. Females may use simple vocal characteristics, such as calling intensity, or male-male interactions. However, it is worth pointing out that pulses are observed in male calls; the calls of females do not contain pulses. As a result, PPD-encoded information, which plays a role in species recognition, may also be used to evaluate sender quality, as is the case in insects [51], [52].

## Supporting Information

Call S1 **Example of natural corncrake call.**
(WAV)

Call S2 **Example of artificial call with the same acoustic energy in each frequency range.** PPDs distribution in the artificial call and in the natural call (Call S1) is the same.
(WAV)

Call S3 **Example of the final artificial call after filtration by graphic equalizer filter.**
(WAV)
